# Supplementary material for: GeneSurfer enables transcriptome-wide exploration and annotation of gene co-expression modules in 3D spatial transcriptomics data
Source: iScience. 2025 Jun 6;28(7):112713. doi: 10.1016/j.isci.2025.112713 (PMC12269424; doi:10.1016/j.isci.2025.112713)
Supplement: Document S1. Figures S1–S3 and Table S1 [file mmc1.pdf]

iScience, Volume *28*

Supplemental information

GeneSurfer enables transcriptome-wide exploration and annotation of gene co-expression modules in 3D spatial transcriptomics data

Chang Li, Julian Thijssen, Thomas Kroes, Ximaine van der Burg, Louise van der Weerd, Thomas Höllt, and Boudewijn Lelieveldt

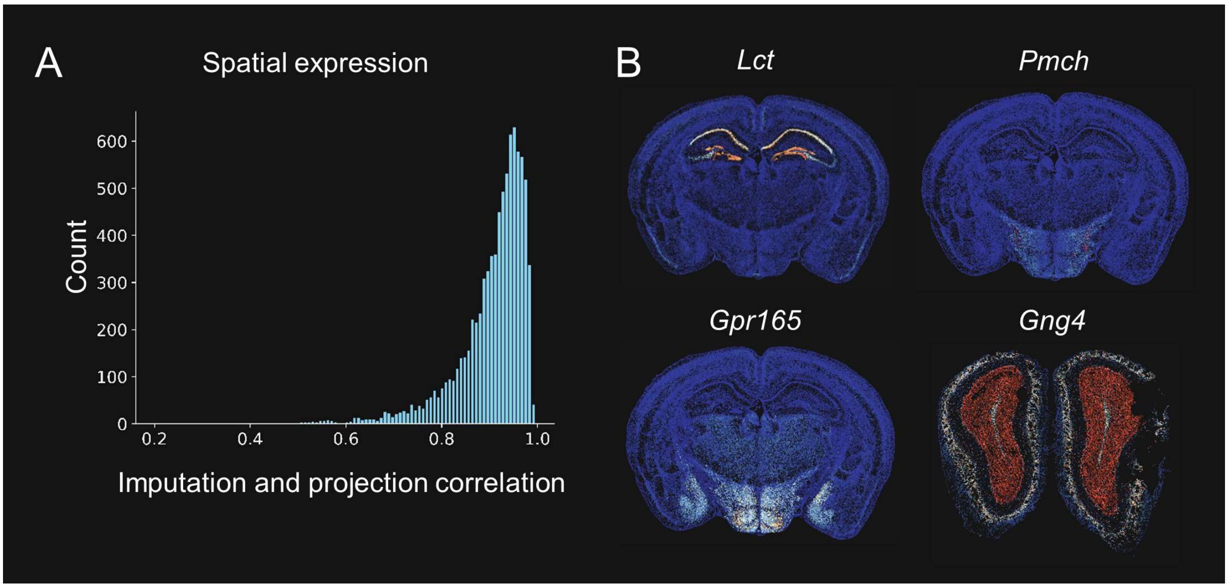

Supplemental Figure 1. Comparison of projection of scRNA-seq annotation averages with the imputed expression from the Allen Institute, related to Figure 2. A. The distributions of Pearson correlation coefficients between the imputed 8,460 genes and scRNA-seq annotation averages across all cells. **B.** Examples of spatial gene expression patterns from imputed data, shown for the same genes as in Figure 2D. A red-to-blue colormap is used, where red indicates high expression, light blue represents low expression, and cells with no expression are shown in dark blue.

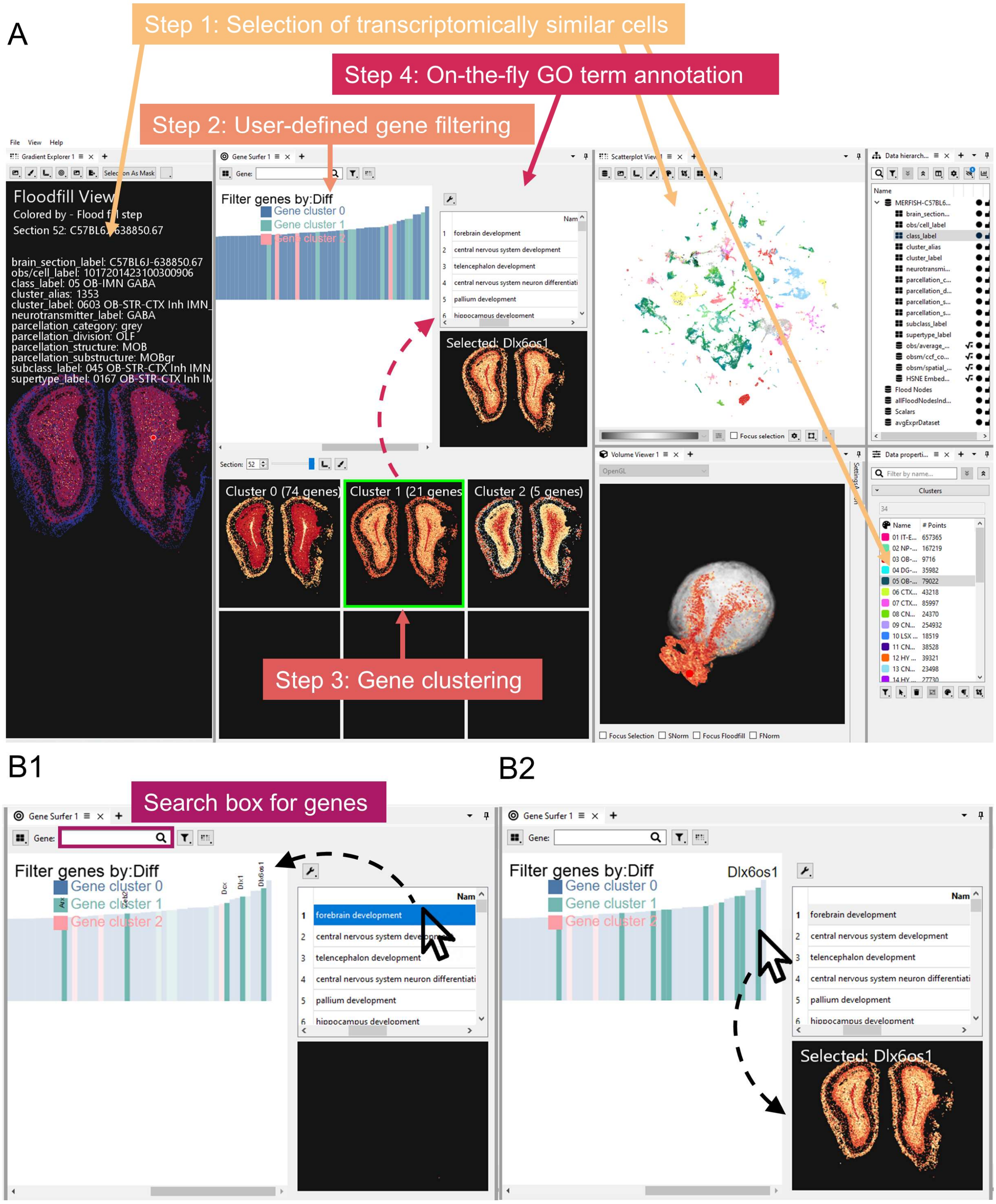

Supplemental Figure 2. GeneSurfer interface and interaction loop, related to STAR METHODS. A. Screenshot of the interface of GeneSurfer with SpaceWalker. **B1.** When the user selects a GO term from the table, associated genes are highlighted in the bar chart. **B2.** Hovering over the bars shows the corresponding gene symbols; simultaneously,

genes within the same cluster as the hovered gene are highlighted. Clicking on a gene displays its expression in both 2D and 3D visualizations.

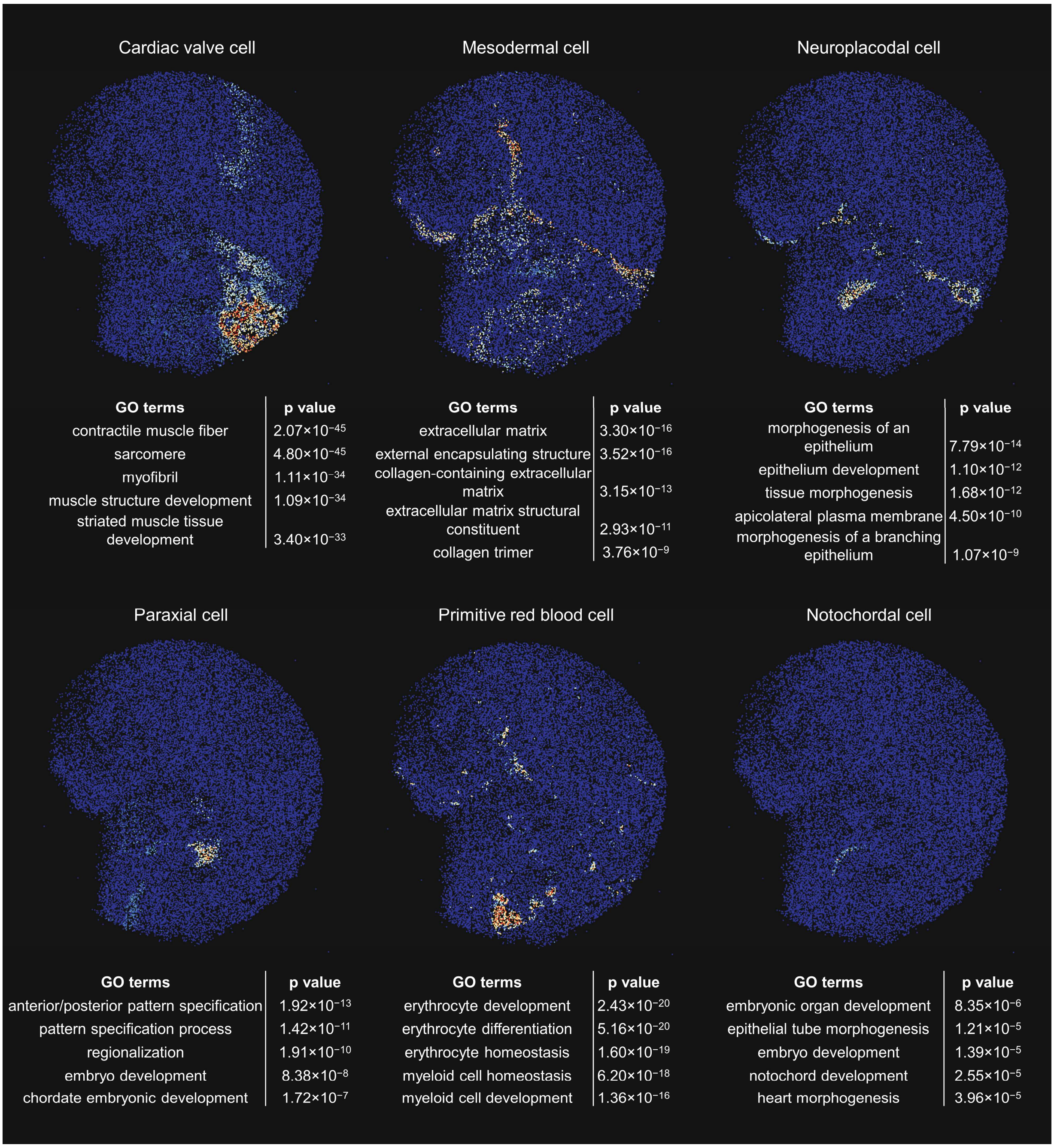

Supplemental Figure 3. Exploration results of Slide-seqV2 mouse embryo data[31] (E9.5:201112_04) using GeneSurfer, related to Figure 4. Cells are selected from the cell type annotations. For each cell type selected from the annotation, the average expression of genes in cluster 0 is shown in the figure. GO terms associated with the filtered genes are retrieved, with the figure presenting the five GO terms having the lowest p-values. GO terms are retrieved by gProfiler with a background gene set (27,554 genes in the dataset). All p-values have been adjusted using the Bonferroni

correction. The gene expression patterns shown are within the selected subset and are color-coded based on normalized values within the subset using a red-to-blue colormap. Red indicates high expression, light blue represents low expression, and cells with no expression are shown in dark blue. (Parameters: differential expression filter, 50 genes, 3 clusters)

Supplemental Table 1. Top 20 differentially expressed genes for each substructure compared to subclass 5310 Endo_NN_1, related to Figure 5. This table lists the top 20 genes that are differentially expressed in each substructure in the HSNE embedding compared to the endothelial subclass. Each column represents one substructure.

1	2	3	4	5	6	7	8	9
Slc17a7	*Gpr88*	*Tcf7l2*	*Agt*	*Pvalb*	*Igf2*	*Gfap*	*Abcc9*	*Abcc9*
Rasgrp1	*Syndig1l*	*Shox2*	*Serpine2*	*Hopx*	*Dcn*	*Aqp4*	*Kcnj8*	*Kcnj8*
Sv2b	*Scn4b*	*Ramp3*	*Syt2*	*Calb1*	*Nr2f2*	*Agt*	*Rgs4*	*Egflam*
Nfix	*Penk*	*Tnnt1*	*Slc17a6*	*Slc38a1*	*Igfbp2*	*Lpar1*	*Egflam*	*Rgs4*
Grm3	*Bcl11b*	*Slc17a6*	*Unc13c*	*Ppp1r17*	*Slc7a11*	*Mog*	*Lhfp*	*Lhfp*
Slc1a3	*Drd2*	*Agt*	*Spon1*	*Shisa6*	*Gfap*	*Sox10*	*Ntn1*	*Ntn1*
Adcy2	*Crym*	*Spock3*	*Slc7a10*	*Kit*	*Egflam*	*Gja1*	*Slc17a7*	*Zic1*
Nrn1	*Spock3*	*Rasgrp1*	*Pvalb*	*Zic1*	*Pcp4l1*	*Hopx*	*Grm3*	*Nr2f2*
Pou3f3	*Rgs4*	*Spon1*	*Zfhx4*	*Unc13c*	*Lama1*	*Zeb2*	*Zic1*	*Igf2*
Ptk2b	*Caln1*	*Grm1*	*Gja1*	*Whrn*	*Fli1*	*Ccdc3*	*Nr2f2*	*Baiap3*
Gda	*Rasgrp1*	*Zic1*	*Aqp4*	*Ctxn3*	*Fxyd6*	*Slc38a1*	*Igf2*	*Gpc3*
Pde1a	*Drd1*	*Rgs4*	*Slc32a1*	*Slc1a3*	*Igfbp4*	*Marcksl1*	*Ror1*	*Agt*
Cxcl14	*Grm3*	*Lhfp*	*Gad2*	*Cnr1*	*Samd5*	*Bmpr1b*	*Car4*	*Ebf1*
Rgs4	*Gad2*	*Adra1b*	*Baiap3*	*Syndig1*	*Ntn1*	*Cdh20*	*Gpc3*	*Car4*
Lrp4	*Syt6*	*Nr2f1*	*Igsf1*	*Cbln1*	*Foxo1*	*Sox2*	*Tbx3*	*Ror1*
St6galnac5	*Slc32a1*	*Samd5*	*Cyp26b1*	*Syt2*	*Lhfp*	*Opalin*	*Ebf1*	*Grm3*
Ccn3	*Unc13c*	*Medag*	*Calb2*	*Grm1*	*Zfp521*	*Klk6*	*Rasgrp1*	*Unc13c*
Ccnd2	*Pcp4l1*	*Sema5a*	*Kcnk9*	*Ntn1*	*Maf*	*Chn2*	*Nr2f1*	*Tmie*
Lamp5	*Six3*	*Fam163a*	*Ramp1*	*Slc32a1*	*Vegfc*	*Prkcq*	*Samd5*	*Pde3a*
C1ql3	*Cnr1*	*Mctp2*	*Gsta4*	*Zfp521*	*Creb3l1*	*Dock5*	*Col27a1*	*Sema5a*
